# SYSTEMATIC EXCLUSION AT STUDY COMMENCEMENT MASKS EARLIER MENOPAUSE FOR BLACK WOMEN IN THE STUDY OF WOMEN’S HEALTH ACROSS THE NATION (SWAN)

**DOI:** 10.1093/geroni/igac059.1862

**Published:** 2022-12-20

**Authors:** Alexis Reeves, Michael Elliott, Carrie Karvonen-Gutierriez, Sioban Harlow

**Affiliations:** Stanford University, School of Medicine, Berkeley, California, United States; University of Michigan, Ann Arbor, Michigan, United States; University of Michigan, Ann Arbor, Michigan, United States; University of Michigan, Ann Arbor, Michigan, United States

## Abstract

Assumptions regarding “normative” aging, including average age at onset of disease, are often based on White populations. However, evidence suggests that Black and Hispanic populations may experience “weathering” or accelerated health declines compared to Whites due to the cumulative impact of social and economic marginalization. If “weathering” leads to a differential probability of inclusion into a cohort study, it will likely misinform understanding of aging in minoritized populations. Using SWAN, a longitudinal multi-ethnic cohort of midlife women, and its cross-sectional screening study, we quantified the extent of potential selection bias at study commencement and re-estimated racial/ethnic differences in age at menopause, the main outcome of SWAN, with adjustment for various forms of potential selection bias. Left truncation was corrected for using inverse probability weighting and right censoring using multiple imputation. Two selection mechanisms were identified, eligibility and participation. Black and Hispanic women had the lowest probability of eligibility stemming from a high prevalence of surgical menopause. Their eligibility rates decreased with increasing age faster than in White women. Correcting for selection biases showed that uncorrected analyses overestimated of the median age of menopause in Black and Hispanic women, thereby underestimating racial/ethnic disparities. After adjustment, Black women had earlier natural and surgical menopause (average 1.2 years) versus White women. Overall, this study found that failure to account for different forms of selection can lead to mis-estimation of racial/ethnic disparities in health and aging. Selection bias is particularly acute at study commencement, and particularly affects minoritized populations.

